# Radial head arthroplasty vs. open reduction and internal fixation in Mason 3 radial head fractures: meta-analysis of prospective trials

**DOI:** 10.1016/j.jseint.2024.08.180

**Published:** 2024-08-27

**Authors:** Domenico De Mauro, Sami Abou Chakra, Francesco Liuzza, Amarildo Smakaj, Giuseppe Rovere, Giulio Maccauro, Omar El Ezzo

**Affiliations:** a Orthopedic Unit, Department of Public Health, Federico II University, Naples, Italy; b Department of Orthopedics and Geriatric Sciences, Catholic University of the Sacred Heart, Rome, Italy; c Orthopedics and Traumatology Unit, Department of Ageing, Orthopedics and Rheumatological Sciences, Fondazione Policlinico Universitario A. Gemelli IRCCS, Rome, Italy; d Orthopedics and Traumatology Unit, Tor Vergata University, Rome, Italy

**Keywords:** Radial head fractures, Radial head arthroplasty, Mason type-3, Surgical treatments, Open reduction and internal fixation, Meta-analysis

## Abstract

**Background:**

Radial head fractures (RHF) represent about one-third of all elbow fractures, comprising approximately 2%-5% of all fractures sustained. The aims of this systematic review and meta-analysis are as follows: (i) to compare complications rate in patients undergoing radial head arthroplasty (RHA) or open reduction and internal fixation as surgical treatments for Mason type 3 RHF; (ii) to compare clinical outcome and functional score in patients undergoing RHA or ORIF in Mason type 3 RHF.

**Methods:**

Following the preferred reporting items for systematic reviews and meta-analyses guidelines, a comprehensive literature systematic review of literature was conducted up to March 2024. All prospective studies were included. The analysis employed the log odds ratio (OR) and 95% confidence interval (CI) as the outcome measure.

**Results:**

Six studies were incorporated into the systematic review. A total of three studies, published between 2009 and 2021, were included in the meta-analysis. A cohort of 169 patients affected by Mason 3 RHFs was collected. The ORIF group included 65 patients, and 26 events of complications after ORIF were observed. RHA group, instead, consisted of 70 patients, and 8 events of complications were identified.

**Conclusion:**

Our findings reveal that the Mason type 3 RHFs treated with open reduction and internal fixation, exhibits a higher risk of complications compared to those patients treated with RHA. Moreover, the standardized mean difference analysis suggests that the ORIF group demonstrates a lower mean Broberg and Morrey Elbow score in comparison to the RHA group, with a higher functional recovery in RHA group.

Radial head fractures (RHFs) represent about one-third of all elbow fractures, comprising approximately 2%-5% of all sustained fractures.^17^^,^^22^^,^^33^ These fractures commonly occur following a fall onto an outstretched arm but can also result from traffic accidents, sports injuries, or anything that involves a forceful collisions between the radial head and capitulum.^18^^,^^27^ Individuals in their 4th and 5th decades of life are at a heightened risk of experiencing such injuries.^16^^,^^18^ Furthermore, RHF occurs slightly more frequently in women, with a 1.4 times higher incidence rate compared to men.^16^, ^17^, ^18^ Beyond the age of 50, it becomes apparent that women are significantly more affected than men.^17^ Speculation suggests that this gender disparity may be attributed to postmenopausal osteoporosis; however, further research is necessary to confirm this hypothesis.^16^ To classify the severity of the fracture, the Mason classification, also known as the Mason-Johnston classification, is utilized. This classification system provides distinct categories for different fracture patterns, guiding appropriate treatment indications.^21^ Mason type 1 fracture is considered the least severe, while Mason type 3 represents the most severe condition being displaced and severely comminuted.^21^ Among these, the most common is the Mason type 1 fracture, constituting 60%-70% of RHF cases characterized by a simple fracture without displacement.^18^ The gold standard treatment for type 1 fractures is nonoperative, typically involving immobilization in a cast for a few days following the trauma.^20^ Type 2 fractures represent a slight worsening from type 1, characterized by minimal displacement (>2 mm).^9^^,^^26^^,^^27^ The treatment for these fractures remains a subject of debate, as evidence supports both conservative and surgical approaches, ultimately leaving the decision to the discretion of the surgeon.^19^^,^^23^^,^^32^ Type 3 fractures pose greater challenges in treatment compared to the previously mentioned types due to the displacement and comminution of the RHF. Displacement can be described as the altering of the radial heads position from its original place and forms a mechanical block to forearm rotation.^1^^,^^12^ Type 4 involves elbow dislocation associated with the RHF, regardless of the severity of the fracture.^14^^,^^27^ While there is no consensus on the standard treatment for type 3 and 4 RHFs, the literature agrees that surgical intervention is the preferred option in these cases.^4^^,^^33^^,^^34^ Regarding surgical techniques for RHF, several options exist as follows: radial head arthroplasty (RHA), open reduction and internal fixation, and radial head excision or resection (RHE). RHE involves the removal of the radial head, which can be criticized due to the importance of radio-capitellar contact for elbow stability.^11^^,^^13^^,^^30^ Patients undergoing radial head excision may potentially experience elbow instability; therefore, it is recommended for less active patients, such as the elderly, or in cases of severely comminuted fractures without associated ligament injury.^13^^,^^15^ Open reduction and internal fixation (ORIF) is recommended when anatomical reduction is achievable.^15^^,^^30^ According to Ring et al,^28^ optimal outcomes with ORIF are observed in cases with no more than 3 fracture fragments as well as slightly comminuted fractures. Radial head arthroplasty (RHA) involves the replacement of the radial head with a prosthesis, typically made of silicone or metal. This procedure is primarily reserved for comminuted fractures, where reduction is not feasible or fractures with 4 or more fragments.^27^ The most common complication associated with RHA is the overstuffing of the radio-capitellar joint, leading to capitellar erosion or elbow stiffness due to the prosthesis being too long.^15^^,^^27^^,^^28^

The aims of this systematic review and meta-analysis are as follows: (i) to compare complications rate and clinical outcomes in patients undergoing RHA or ORIF as surgical treatments for Mason type 3 RHF; (ii) to review systematically prospective studies in the literature, analyzing different treatment options for Mason type 2 and 3 RHFs.

## Materials and methods

### Search strategy and eligibility criteria

Following the preferred reporting items for systematic reviews and meta-analyses guidelines,^25^ a comprehensive literature systematic review of literature was conducted up to March 2024. This review specifically focused on randomized controlled trials (RCTs) and prospective studies comparing clinical outcomes and complication rates in patients with type 2 and 3 RHFs, according to the Mason classification.^21^ The search strategy included three prominent online databases as follows: MEDLINE, Google Scholar, and Scopus. Keywords used were combined as follows: “RHFs” or “RHF” and “treatment” or “comparison” or “RHA” or “ORIF”, along with relevant Medical Subject Headings combinations.

All prospective studies written in English were included without restrictions on publication date. Additional articles not found in the initial search were identified by thoroughly examining the reference lists of selected articles. Longitudinal retrospective studies, although comparing treatment options for RHFs, were excluded from the review. Included studies underwent thorough evaluation and were subsequently added to the final reference list for the systematic review. Meta-analysis was conducted only on prospective studies comparing the ORIF group and the RHA group due to limited data available for evaluating other treatment options.

The inclusion criteria were as follows: (i) prospective trials including (ii) patients aged 18 years or older and (iii) diagnosed with Mason type 2 and type 3 RHFs.

The exclusion criteria were as follows: (i) case reports, expert opinions, previous systematic reviews, letters to the editor, retrospective studies; (ii) studies that did not assess complications or clinical outcomes; (iii) studies with incomplete data.

### Study assessment and data extraction

Initially, two independent reviewers (O.E.E and S.A.C) screened the titles and abstracts of the studies. Full texts were obtained for all abstracts that appeared to meet the inclusion criteria or presented any uncertainty. Subsequently, two independent reviewers (D.D.M and A.S) scrutinized each study based on the inclusion criteria. Any discrepancies in inclusion were resolved through assessment by the senior author (F.L and G.M). Relevant data, including participant demographics, sample size, surgical data, outcomes, and complications, were systematically extracted from each study. A comparative meta-analysis was conducted focusing on the declared endpoints, which included: (i) a comparison of clinical outcomes between the ORIF group and the RHA group and (ii) a comparison of complications between these groups. Clinical outcomes were assessed using the Broberg and Morrey Elbow score (BMES),^2^ with reported mean and standard deviation values.

The methodological quality of the studies included in this meta-analysis was assessed using the modified Coleman Methodology Score (mCMS).^8^ Two authors (F.L and G.R) independently evaluated the mCMS for each study, and any discrepancies were resolved through consensus to derive the final score.

### Statistical analysis

The analysis employed the log odds ratio (OR) and the standardized mean difference (SMD) and 95% confidence interval (CI) as the outcome measure. Due to the expected diversity among the studies included, a random-effects model was utilized for data fitting. The degree of heterogeneity (τ^2^) was assessed using the restricted maximum-likelihood estimator.^37^ Furthermore, the analysis includes the Q-test for heterogeneity and the I^2^ statistic.^7^ If any level of heterogeneity is detected (τ^2^ > 0), a prediction interval for the true outcomes is provided. Studentized residuals and Cook's distances were utilized to evaluate whether studies could potentially be outliers and/or exert influence within the model framework. Studies with a studentized residual exceeding the $\left\{100\times \left[1\;-\frac{0.05}{\left(2k\right)}\right]\right\}$ ^th^ percentile of a standard normal distribution were deemed potential outliers. This classification applied a Bonferroni correction, adopting a two-sided significance level of α = 0.05 for k studies included in the meta-analysis. Studies with a Cook's distance, exceeding the median plus six times the interquartile range of the Cook's distances, were identified as influential. Funnel plot asymmetry was assessed using both the rank correlation test and the regression test, with the standard error of the observed outcomes used as the predictor. The pooled incidence of complications was calculated using dichotomous models and reported as odds ratios (OR) with corresponding 95% confidence intervals (CI). For the analysis of clinical scores, SMD was utilized as the outcome measure and reported as odds ratios (ORs), with corresponding 95% CIs. Statistical analyses were performed using SPSS version 29 (IBM Corp., Armonk, NY, USA). A significance level of *P* ≤ .05 was considered significant.

## Results

### Search and selection process

**Figure 1:**
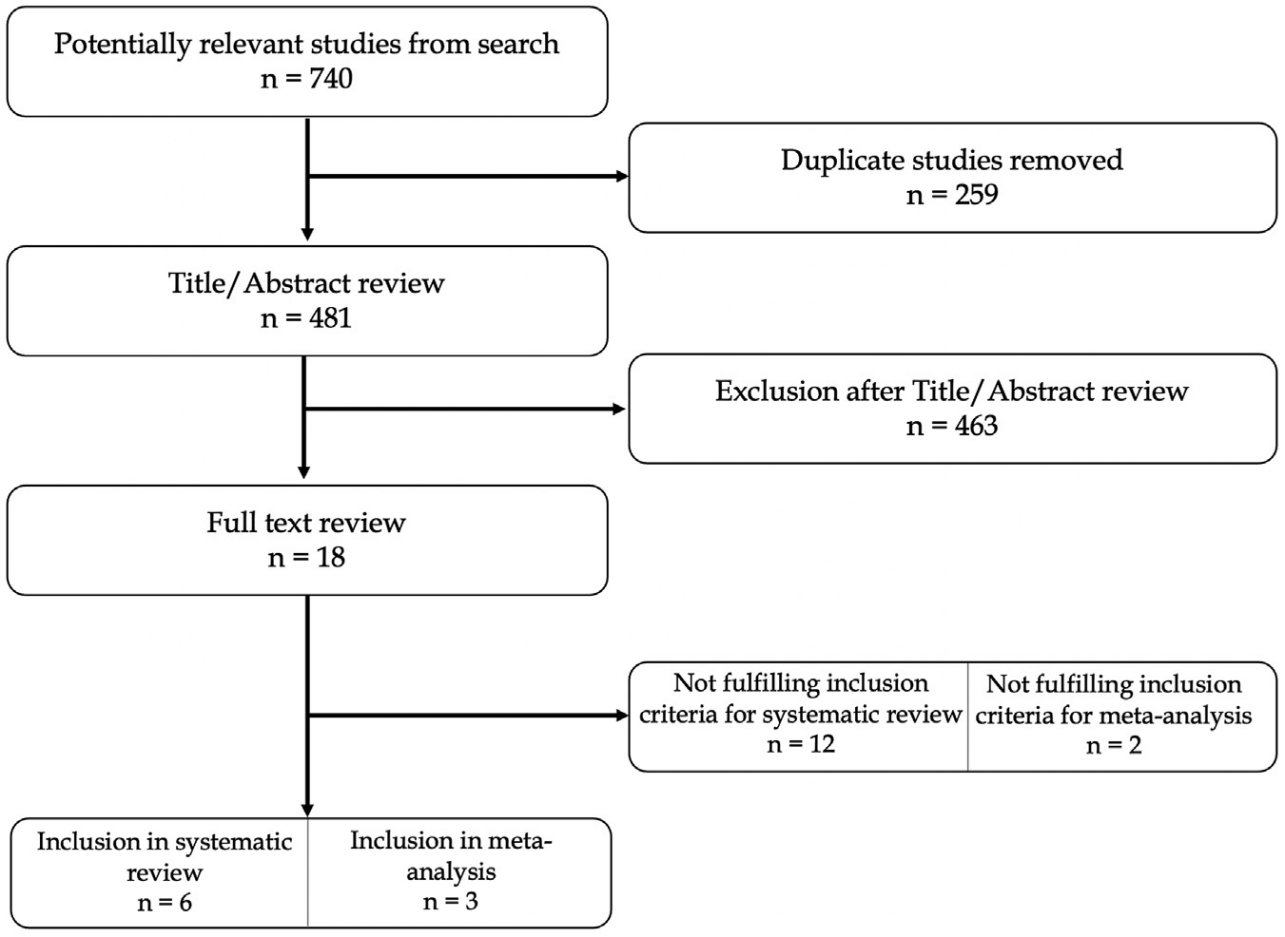
PRISMA flowchart. *PRISMA*, preferred reporting items for systematic reviews and meta-analyses.

**Figure 2:**
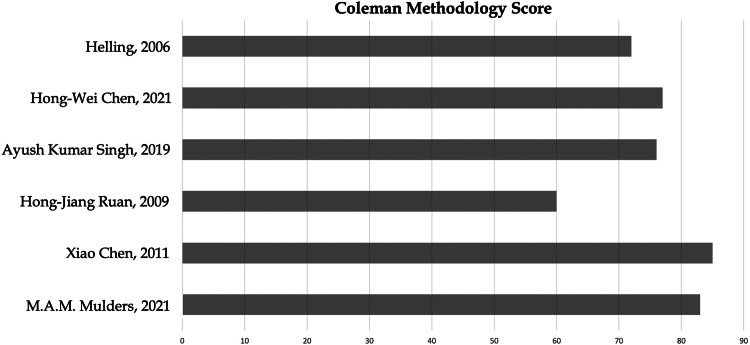
Coleman methodology Score of the included studies.

The study flow chart is presented in Figure 1. The initial literature search yielded 740 articles. Duplicates were subsequently removed, and following this initial step, the remaining papers underwent screening based on titles and abstracts. After excluding case reports, expert opinions, previous systematic reviews, letters to the editor, and retrospective studies, the full texts of 18 articles were further assessed for eligibility. Through this full-text analysis, no additional articles were found, sourced from references in the full-text papers admitted for analysis. Following the comprehensive search, papers not meeting the inclusion criteria were excluded, along with those for which corresponding authors did not provide additional data after an official request by mail. Ultimately, 6 papers were included in this systematic review.^5^^,^^6^^,^^10^^,^^23^^,^^29^^,^^31^ Notably, three studies were excluded from the meta-analysis due to evaluations of treatment options different from ORIF and RHA, rendering it unsuitable for data pooling and incongruent with the meta-analytic strategy.^10^^,^^23^^,^^31^ The quality analysis of the included studies was assessed through Coleman score. The results are shown in Figure 2.

### Descriptive data of included studies

Six studies were incorporated into the systematic review^5^^,^^6^^,^^10^^,^^23^^,^^29^^,^^31^; relevant data are collected in Table I.

**Table I:** Patients characteristics from included studies in the systematic review.

First author, year	Study span (y)	Patients (n)	Male %	Age (y)	ORIF (n)	RHA (n)	Resection (n)	Non-op (n)
Mulders et al,^23^ 2021	54	45	44.0	50.0	23	0	0	22
Chen et al,^6^ 2011	60	45	76.0	37.0	23	22	0	0
Helling et al,^10^ 2006	54	135	64.3	39.0	135∗	0	0	0
Ruan et al,^29^ 2009	48	22	59.1	38.4	8	14	0	0
Chen et al,^5^ 2021	102	102	52.9	36.1	34	34	34	0
Singh et al,^31^ 2019	36	32	59.4	43.4	0	15	17	0

*ORIF*, open reduction internal fixation; *RHA*, radial head arthroplasty; *Non-op*, nonoperative.

∗ Comparison between two different devices in ORIF.

The population sizes varied significantly among the studies, ranging from 22^29^ to 135^10^ patients with Mason 2 or 3 RHF. The mean age across the studies ranged from 36.1 to 43.4 years, with an overall mean age of 38.8 ± 2.8 years. One of the studies^23^ express the age value with median and interquartile range (IQR) of 50 and 46-58, respectively.

The majority of participants were male, constituting 59.3% of the patients. The mean follow-up ranged from 12 to 30 months, with an overall mean of 18.0 ± 5.8 months. About treatment, three studies compared ORIF with RHA, and^5^^,^^6^^,^^29^ one study compared, instead, ORIF and conservative treatment.^23^ One study^10^ compared two different devices in ORIF, and one last study, finally, compared RHA with radial head resection^31^ (Table I). As matter of clinical evaluation of elbow function, Mayo Elbow Performance Score (MEPS)^35^ was used by two studies,^23^^,^^31^ and in four studies, instead, BMES was used.^2^^,^^5^^,^^6^^,^^10^^,^^29^

### ORIF vs. RHA in Mason type- 3 RHFs: meta-analysis

A total of three studies, published between 2009 and 2021, compared Mason 3 RHF treated with ORIF and RHA, and therefore were included in the analysis. A cohort of 169 patients affected by Mason 3 RHFs was collected. Demographic data and patient characteristics are summarized in Table II.

**Table II:** Collected data from included prospective trials in the meta-analysis between ORIF and RHA in Mason 3 fractures.

First author, year	Patients (n)		Mason∗	Follow-up (mo)	Complication(s) (n)		BMES (mean, SD)	
	ORIF	RHA			ORIF	RHA	ORIF	RHA
Chen et al,^6^ 2011	23	22	3	24.0	11	3	72.4 ± 7.1	92.1 ± 6.8
Ruan et al,^29^ 2009	8	14	3	15.2	7	3	69.6 ± 12.5	92.6 ± 9.7
Chen et al,^5^ 2021	34	34	3	12.0	8	2	77.8 ± 7.5	83.1 ± 8.6

*ORIF*, open reduction internal fixation; *RHA*, radial head arthroplasty; *BMES*, Broberg and Morrey Elbow score; *SD*, standard deviation.

∗ Mason Classification.

The ORIF group comprised 65 patients, with a mean age of 37.5 ± 3.6 years and a male ratio of 54.8%. Within this group, 26 events of complications after ORIF were observed.

Conversely, the RHA group also consisted of 70 patients, with a mean age of 37.5 ± 0.1 years and a male ratio of 56.5%. In this group, 8 events of complications after RHA were identified. Complications are shown in Table III. Two different subanalysis were carried out: (i) complications rate and (ii) clinical outcomes were meta-analyzed among the two different groups, ORIF and RHA, respectively.

**Table III:** Complications from included prospective trials in the meta-analysis between ORIF and RHA in Mason 3 fractures.

First author, year	Non-union (n)		Infection (n)		HO (n)		Stiffness (n)		Others∗ (n)	
	ORIF	RHA	ORIF	RHA	ORIF	RHA	ORIF	RHA	ORIF	RHA
Chen et al,^6^ 2011	4	0	1	0	2	0	0	1	4	2
Ruan et al,^29^ 2009	4	0	0	0	0	3	0	0	3	0
Chen et al,^5^ 2021	0	0	0	0	1	1	4	1	3	0

*HO*, Heterotopic Ossification; *ORIF*, open reduction internal fixation; *RHA*, radial head arthroplasty.

∗ Included: Post-traumatic Osteoarthritis, Elbow’s Range of Motion limitation, K-wires loosening.

**Figure 3:**
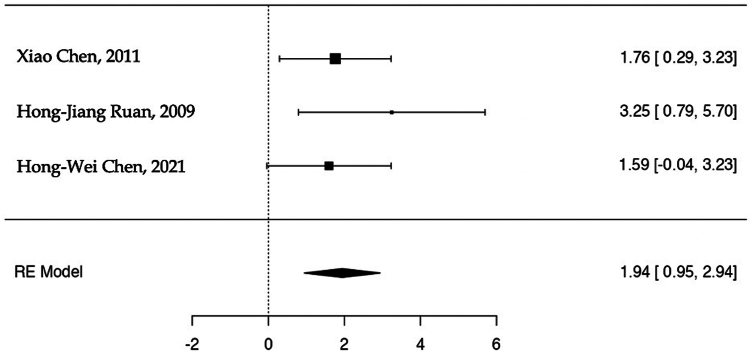
Complication rates in the ORIF and RHA groups are illustrated in the forest plot. A positive OR indicates that the experimental group (ORIF) has a higher risk compared to the control group (RHA). The calculations presented were derived using random-effects models, and the confidence intervals are depicted by the bars in the graph. *ORIF*, open reduction internal fixation; *RHA*, radial head arthroplasty.

**Figure 4:**
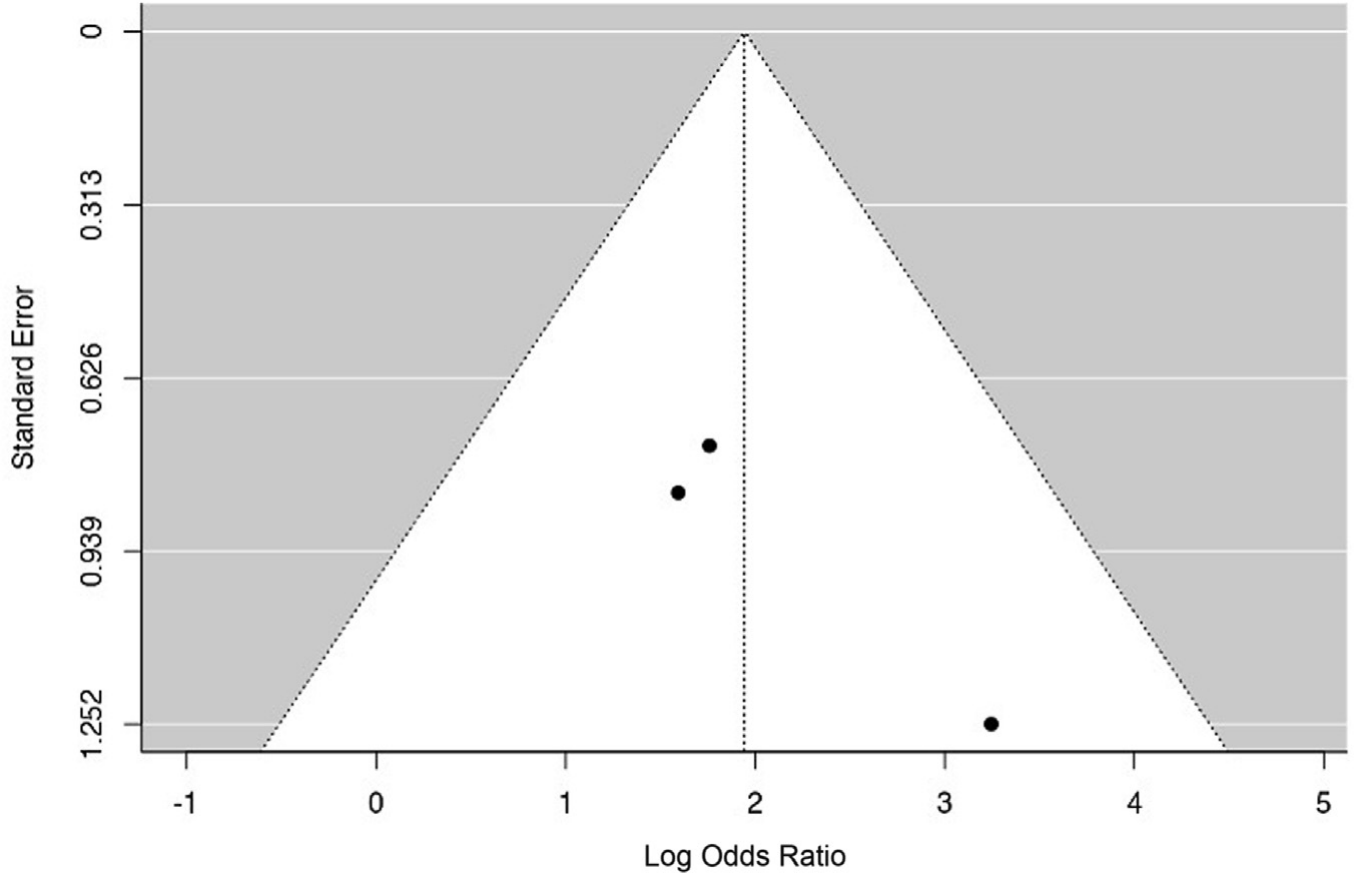
The funnel plot depicting the meta-analysis of complications in ORIF vs. RHA showed that none of the studies exerted excessive influence. Additionally, both the rank correlation and regression tests indicated no evidence of asymmetry in the funnel plot. *ORIF*, open reduction internal fixation; *RHA*, radial head arthroplasty.

For the first sub-analysis (i), 34 events were analyzed (complications after ORIF or RHA). The estimated average log OR based on the random-effects model was OR: 1.94 (95% CI: 0.95-2.94). Therefore, the average outcome differed significantly from zero (z = 3.81, *P* = .0001). A positive OR indicates that the experimental group (ORIF) has a higher risk compared to the control group (RHA). According to the Q-test, there was no significant amount of heterogeneity in the true outcomes (Q(2) = 1.318, *P* = .517, tau^2^ = 0.000, I^2^ = 0.000%). The relative Forest plot is shown in Figure 3. An examination of the studentized residuals revealed that none of the studies had a value larger than ± 2.3940, and hence there was no indication of outliers. Neither the rank correlation nor the regression test indicated any funnel plot asymmetry (*P* = 1.000 and *P* = .280, respectively) (Fig. 4).

For the second sub-analysis, (ii) clinical outcomes were assessed. All studies included in the analysis utilized the BMES, presenting mean results along with standard deviations. Therefore, SMD were employed.

**Figure 5:**
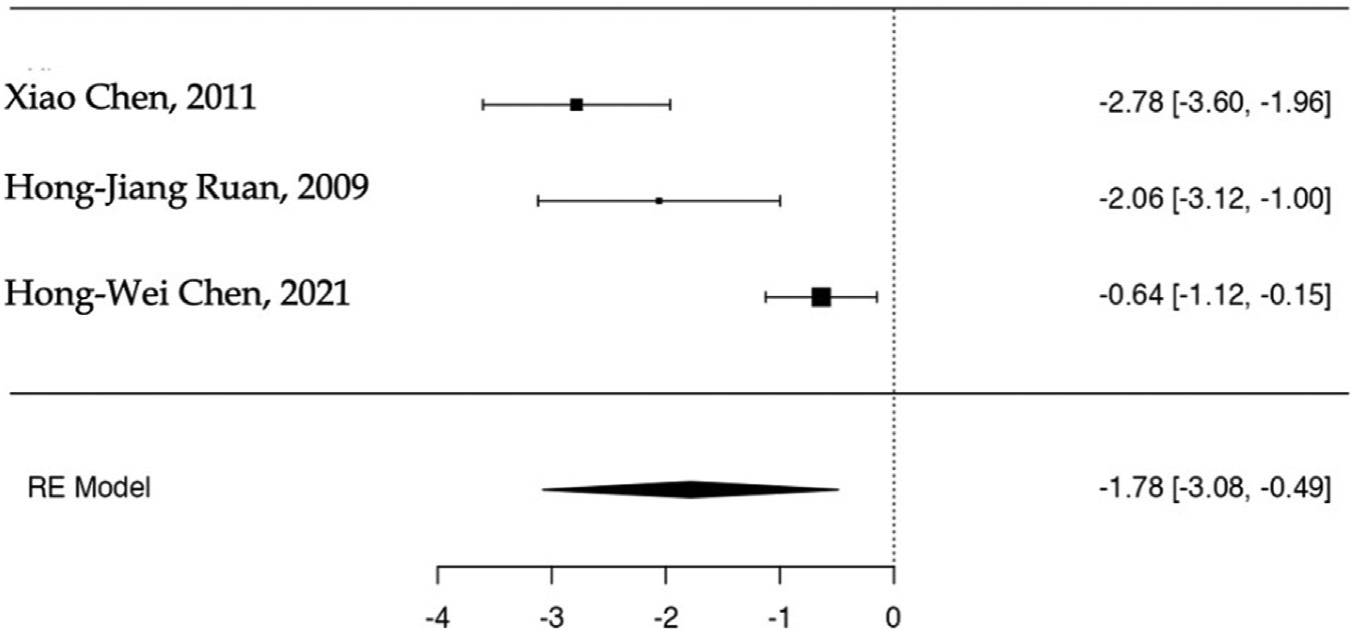
Clinical outcomes in the ORIF and RHA groups are illustrated in the forest plot. A negative standardized mean difference (SMD) indicates that the experimental group (ORIF) has a lower mean score compared to the control group (RHA). The calculations presented were derived using random-effects models, and the confidence intervals are depicted by the bars in the graph. *ORIF*, open reduction internal fixation; *RHA*, radial head arthroplasty.

**Figure 6:**
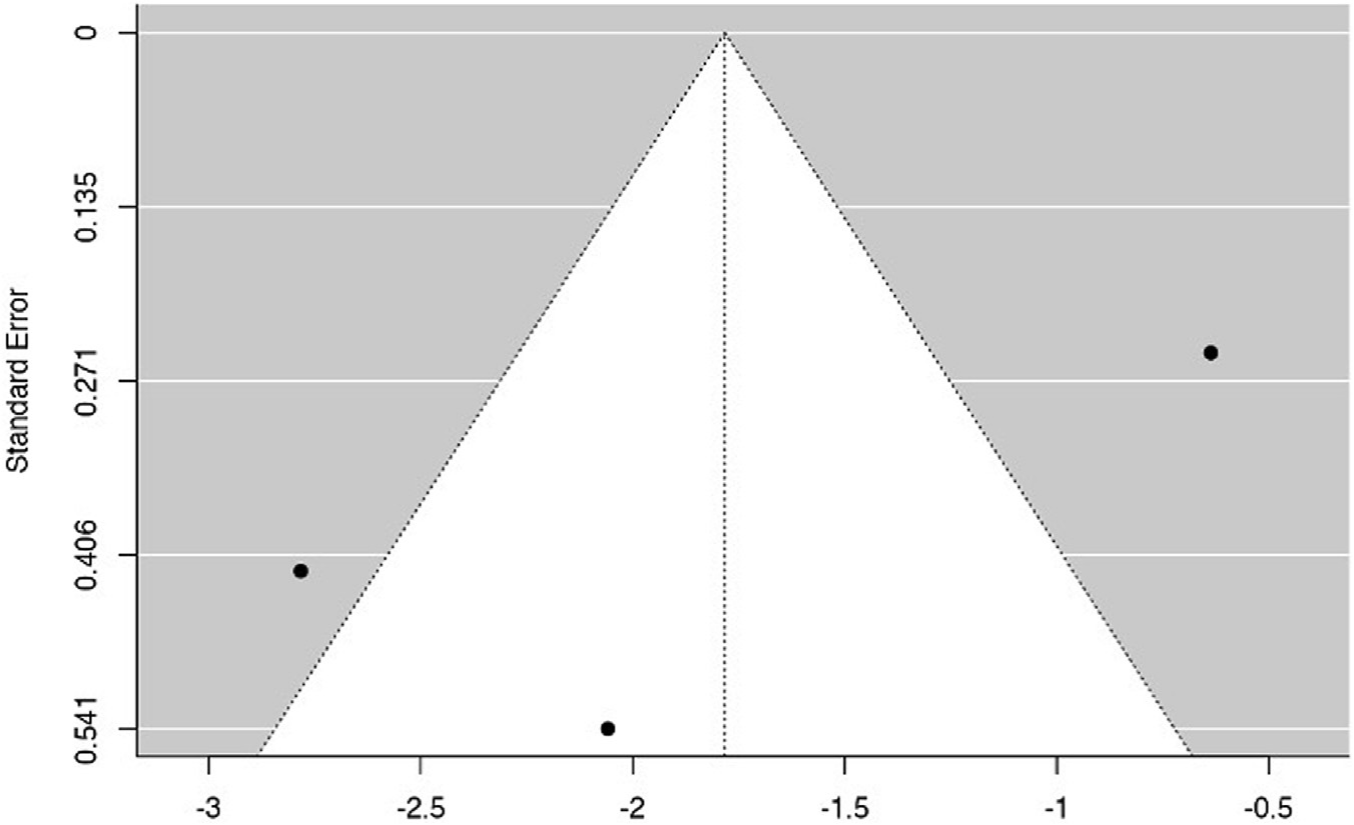
The funnel plot depicting the meta-analysis of clinical outcomes in ORIF vs. RHA showed that none of the studies exerted excessive influence. X-axis represents standardized mean difference, y-axis the standard error, instead. Neither the rank correlation nor the regression test indicated any funnel plot asymmetry (*P* = 1.000 and *P* = .270, respectively). *ORIF*, open reduction internal fixation; *RHA*, radial head arthroplasty.

The estimated average SMD based on the random-effects model was −1.78 (95% CI: −3.08 to −0.49). Hence, the average outcome significantly deviated from zero (z = −2.697, *P* = .007). A negative SMD indicates that the experimental group (ORIF) has a lower mean score compared to the control group (RHA). The Q-test indicated significant heterogeneity among the true outcomes (Q(3) = 21.638, *P* < .0001, tau^2^ = 1.141, I^2^ = 88.3%) (Fig. 5). An examination of the studentized residuals revealed that one of the studies had a value larger than ± 2.49 and hence there was outlier in the context of this model.^5^ According to the Cook's distances, none of the studies could be considered to be overly influential. Neither the rank correlation nor the regression test indicated any funnel plot asymmetry (*P* = 1.000 and *P* = .27, respectively) (Fig. 6).

## Discussion

A comprehensive literature review was conducted to identify RCTs and prospective trials focusing on the surgical management of RHFs. The scope of the review naturally centered on studies evaluating operative interventions for Mason type 2 and type 3 fractures, as Mason type-1 fractures typically receive nonoperative management. The objective of the meta-analysis, in particular, was to analyze high-quality studies to compare RHA and ORIF as treatment in Mason 3 fractures and provide an updated synthesis of the literature on this subject matter.

The main findings of our study reveal significant differences in complication rates between patients undergoing ORIF vs. RHA. Particularly noteworthy is the higher risk of complications associated with ORIF compared to RHA. Across the three meta-analyses included in our study, the mean complication rate was found to be 52.9% in the ORIF group and 13.6% in the RHA group. In the study conducted by Xiao Chen et al,^6^ the complication rate was 47.8% in the ORIF group and 13.6% in the RHA group, further emphasizing the disparity in complication rates between the two treatment modalities. Similarly, Hong-Jiang Ruan et al,^29^ the complication rate was 87.5% in the ORIF group and 21.4% in the RHA group. Moreover, the ORIF group in the Hong-Wei Chen et al^5^ also recorded a larger complication rate compared to the RHA group (23.5% and 5.9%).

Another significant finding from our meta-analysis pertains to the clinical outcomes, as measured by the BMES. Our analysis revealed that the RHA group consistently exhibited higher BMES values compared to the ORIF group, indicating superior functional recovery in patients undergoing RHA. Across all three studies included in the meta-analysis, the mean BMES values were consistently higher for the RHA group relative to the ORIF group.^5^^,^^6^^,^^29^ Notably, the differences in BMES values between the two groups were found to be statistically significant (*P* < .05) in all studies. Xiao Chen et al^6^ reported a mean difference of 19.7 (92.1 vs. 72.4) in BMES values, while Hong-Jiang Ruan et al,^29^ reported a mean difference of 23.0 (92.6 vs. 69.6), and Hong-Wei Chen et al,^5^ reported a mean difference of 7.7 (94.2 vs. 86.5), all favoring RHA over ORIF. These findings underscore the superior functional outcomes associated with RHA compared to ORIF, providing valuable insights for clinical decision-making in the management of RHFs.

The mCMS serves as a critical tool for evaluating the quality of clinical studies, offering a systematic assessment of the methodologies employed in research trials. This score, ranging from 0 to 100, provides valuable insights into the reliability of study methods. In our systematic review, comprising six studies, the average mCMS was calculated to be 75.5, indicating a robust methodology across the reviewed trials. Similarly, the meta-analysis, including three studies, reached an average mCMS of 74.0. Notably, both the systematic review and the meta-analysis received a favorable grade, falling within the range of 70-84, indicative of good methodological practices and robust study designs. These findings underscore the overall quality and reliability of the reviewed studies, enhancing the credibility and validity of the conclusions drawn from our research.

Mulders et al^23^ conducted a multicenter RCT assessing surgical (ORIF) vs. conservative treatments of Mason type 2 RHFs in adults. Clinical assessment was performed 3, 6, and 12 months after the intervention. At 12 months, both groups had a median MEPS of 100, indicating the effectiveness of both treatments, with no statistically significant difference between groups (*P* = .28). The complication rates were minimal for both groups, without any considerable differences between treatment options, concluding that the clinical outcome for the treatment of Mason 2 RHF with surgery or a cast is comparable. Singh et al^31^ carried out a randomized prospective comparative study comparing the results of 32 patients, with a Mason type 2 and type 3 RHF treated with replacement or excision of the radial head (RHA vs. RHE). The average follow-up was 20 months, and the MEPS was taken at 6 and 18 months, postoperatively. The excision yielded better results over arthroplasty, with the mean MEPS of 85.7 and 68.8 for the RHE and RHA groups, respectively, at 6 months. At 12 months, the average MEPS was 90.7 for the RHE group and 75 for the RHA group. The two pairs of results showed a statistically significant difference, with a *P* value below .01 for both 6 and 18 months. There was a lower complication rate for the RHE group (23.5% for the RHA group, 0% for the RHE group), excluding pain and infections. To conclude, this trial claims that excision is more effective and yields better results over arthroplasty of the radial head, with fewer complications.

Helling et al^10^ conducted an RCT to evaluate the efficacy of biodegradable polylactide implants compared to standard metal implants in ORIF surgery for displaced RHFs. Among the 184 patients enrolled in the study, only 135 were successfully followed-up at the two-year mark. Polylactide group exhibited a mean BMES of 93.3, while the metal group demonstrated a mean BMES of 90.9 but without significance (*P* = .175), indicating comparable functional outcomes between the two implant types. Moreover, complication rates were similar across both groups. These findings suggest that biodegradable polylactide implants can be considered a viable alternative to standard metal implants.

This meta-analysis provides a higher level of evidence concerning the topic of whether ORIF or RHA yields better results for the treatment of Mason type 3 fractures of the radial head. There are only two systematic review and meta-analysis regarding surgical treatments of RHFs in the last 10 years.^3^^,^^36^ Chaijenkij et al^3^ included 13 studies in the meta-analysis, one was an RCT, and 12 retrospective studies were analyzed. However, it should be clarified that the results of the level 1 and 2 studies are significantly more reliable than the results of retrospective studies considering the biases they may have. Vannabouathong et al,^36^ on the other hand, conducted a meta-analysis similar to ours, encompassing three prospective studies. However, their analysis differed in terms of treatment inclusivity, as they incorporated heterogeneous treatment modalities. In contrast, our study maintained a more homogeneous approach, focusing exclusively on patients undergoing the same surgical procedure for the same diagnosis.

All three studies used in the meta-analysis^5^^,^^6^^,^^29^ were from China, which means that the results have the potential to be misleading since the trials are assessing one specific population, and it does not necessarily represent the rest of the world. Different locations lead toward a possible bias in patients’ selection and treatment options. A study carried out by Casey M. O’Connor et al,^24^ analyzed the factors affecting surgeons likelihood to perform RHA over ORIF, via questionnaires. The geographical location was also a factor, with North American surgeon’s being 9.7 times as likely (*P* < .001) to recommend RHA compared to European surgeons. Preservation of the elbow’s native anatomy seems to be more of a concern for younger patients as well as practice locations outside of North America.

This study presents several limitations. Firstly, as previously mentioned, our study is subject to geographical bias, with a majority of included studies originating from a specific region. Moreover, the small number of studies included in our analysis, along with the consequent limited population size, poses a challenge in drawing definitive conclusions. Despite these limitations, it is worth noting that our study stands as the sole meta-analysis dedicated exclusively to this topic, focusing specifically on prospective studies. Furthermore, our analysis evaluates homogeneous clinical and functional outcomes, providing a comprehensive understanding of the clinical outcome. Additionally, the mean modified Coleman Methodology Score obtained in our study suggests a high level of methodological rigor and robustness, further reinforcing the credibility of our findings. Our study contributes valuable insights to the existing literature, highlighting the need for further research to address these constraints and validate our findings in diverse populations.

## Conclusion

Our findings reveal that the Mason 3 RHFs treated with ORIF, exhibits a higher risk of complications compared to those patients treated with RHA. Moreover, the SMD analysis suggests that the ORIF group demonstrates a lower mean BMES in comparison to the RHA group, with a higher functional recovery in RHA group. This evidence helps to find a proper indication for Mason 3 fractures, considering possible outcomes of both treatments. Further research and clinical trials may be needed to validate these findings and perform evidence-based treatment decisions.

## Disclaimers:

Funding: The authors declare that no funds, grants, or other support were received during the preparation of this manuscript.

Conflicts of interest: The authors, their immediate families, and any research foundation with which they are affiliated have not received any financial payments or other benefits from any commercial entity related to the subject of this article.
